# Developing consensus on core outcome domains and measurement instruments for assessing effectiveness in perioperative pain management after sternotomy, breast cancer surgery, total knee arthroplasty, and surgery related to endometriosis: The IMI-PainCare PROMPT protocol for achieving a consensus on core outcome domains

**DOI:** 10.1186/s13063-020-04665-9

**Published:** 2020-09-10

**Authors:** Ulrike Kaiser, Hiltrud Liedgens, Winfried Meissner, Claudia Weinmann, Peter Zahn, Esther Pogatzki-Zahn

**Affiliations:** 1 Comprehensive Pain Center, University Hospital Carl Gustav Carus Dresden, Dresden, Germany; 2 Market Access, Grünenthal GmbH, Aachen, Germany; 3 Department of Anaesthesiology and Intensive Care Medicine, Jena University Hospital, Jena, Germany; 4 Department of Anaesthesiology and Intensive Care Medicine, Palliative Care Medicine and Pain Management, Berufsgenossenschaftliches Universitätsklinikum Bergmannsheil GmbH Bochum, Ruhr University Bochum, Bochum, Germany; 5 Department of Anaesthesiology, Intensive Care and Pain Medicine, University Hospital Münster, Albert-Schweitzer-Campus 1, A1, 48149 Münster, Germany

## Abstract

**Introduction:**

Evidence synthesis of clinical trials requires consistent outcome assessment. For pain management after surgery, inconsistency of effectiveness assessment is still observed. A subproject of IMI-PainCare (Innovative Medicine Initiatives, www.imi-paincare.eu) aims for identifying core outcome domains and measurement instruments for postoperative pain in four surgical fields (sternotomy, breast cancer surgery, total knee arthroplasty, and surgery related to endometriosis) in order to harmonize outcome assessment for perioperative pain management.

**Methods:**

A multifaceted process will be performed according to existing guidelines (Core Outcome Measures in Effectiveness Trials (COMET), COnsensus-based Standards for the selection of health Measurement INstruments (COSMIN)). In a first step, outcome domains will be identified via systematic literature review and consented on during a 1-day consensus meeting by 10 stakeholder groups, including patient representatives, forming an IMI PROMPT consensus panel. In a second step, outcome measurement instruments regarding the beforehand consented core outcome domains and their psychometric properties will be searched for via systematic literature review and approved by COSMIN checklist for study quality and scale quality separately. In a three-step online survey, the IMI PROMPT consensus panel will vote for most suitable measurement instruments. The process is planned to be conducted between 11/2017 (systematic literature review on common outcome domains) and 3/2022 (final voting on core outcome measurement).

## Introduction

More than 300 million patients receive surgery each year worldwide, where pain is one of the most common and devastating symptoms thereafter [1]. Acute postoperative pain does not only cause suffering in patients for several days; high pain scores early after surgery are associated with postoperative complications like ileus, gastroparesis, constipation, atelectasis, respiratory insufficiency, urinary retention, and thrombosis [2], some with long-term consequences including prolonged, persistent pain for years after surgery [3]. Although efforts to improve the situation of patients have been undertaken, pain management within the first days after surgery is still insufficient [4–7].

Pain management options need to be evaluated regarding their effectiveness in preventing and managing acute postoperative pain [8]. There are several reasons for non-satisfying acute pain management; one of them being a flaw in designing RCTs by choosing study endpoints not displaying clinically relevant treatment effects [9]. Acute pain ratings at rest serve as a common primary outcome [10]; yet, pain intensity at rest is usually less intense than, for example, pain during movement, and does poorly correspond to postoperative rehabilitation (physiotherapy), recovery, length of hospitalization, and long-term consequences including chronic postoperative pain [3, 9]. In fact, it is unclear to date if pain intensity ratings are relevant measures after surgery [2, 11]. The lack of clinically relevant and standardized patient-reported outcome measures (PROMs) for studies addressing the management of postoperative pain impedes identification of effective treatments for certain surgical procedures. Improving comparability of effectiveness research therefore requires a core set of outcome measures in clinical practice and controlled trials for perioperatively managing pain after surgical procedures.

Core outcome sets (COS) are considered legitimate approaches to overcome irrelevant and inconsistent outcome assessment in clinical trials [12]. They are defined “as minimum core sets consisting of patient relevant or reported outcome domains and corresponding measurement instruments to be assessed in any clinical trial regarding a specific health condition and/or intervention” [12]. Outcome domains are defined as concepts to be measured in terms of a further specification of an aspect of health [13], e.g., health-related quality of life. A COS commonly includes patient-reported outcomes (PRO) and patient-reported outcome measures (PROM), the latter understood as “any report of the status of a patient’s health condition that comes directly from the patient, without interpretation of the patient’s response by a clinician or anyone else” [14] and is therefore different from other, so-called “objective” measures such as biomarkers.

The development of such a COS, comprising of both outcome domains and measurement instruments, is a multifaceted process, containing systematic research and consensus processes. Standards for their development have been set by COMET (*Core Outcome Measures in Effectiveness Trials* [12]). Four key features are required in order to establish an accepted and ready to use COS for the research field of concern: structured procedure (e.g., guided by COMET handbook), transparency of performance (e.g., guided by COS Star guidelines of reporting COS studies), transparency of decision criteria, and inclusion of relevant stakeholders (including patient representatives).

The a priori defined health condition *acute postoperative pain* has not been considered as a separate health condition so far. Postoperative pain has been acknowledged as one domain (out of many) in perioperative medicine [15] but was not further established. For total knee arthroplasty (and for knee replacement or joint replacement), several initiatives work on harmonizing outcome assessment [11, 16–28], by considering effects of surgery and general long-term features without focusing on acute postoperative pain (e.g., knee injury and/or knee osteoarthritis, knee, hip, and hand osteoarthritis [16–20, 22–28] or hip or knee osteoarthritis [11, 21]). Regarding breast surgery and sternotomy, COS considerations for postoperative pain have not been worked on yet, despite some effort to harmonize outcome assessment in general for reconstructive breast surgery [29, 30]. Some initiatives have worked on COS for endometriosis [31–34], but again, postoperative pain was not addressed. For all surgical procedures, perioperative pain management is characterized by a short duration of intervention (regularly for some days up to 1 or 2 weeks after surgery), and ideally supporting quick recovery and regain of functioning. Therefore, perioperative pain management aims for enabling the patient to return quickly to as much self-management ability and reduced pain-related interference of wellbeing as possible, depending on limitations of functioning due to the surgical intervention.

PROMPT (Patient-Reported Outcome Measures in Pain Treatment) is one of three subprojects within the IMI-2 JU project IMI-PainCare (Innovative Medicines Initiative Pain Care, www.imi-paincare.eu, 30th March 2020) funded by the European Union and European Federation of Pharmaceutical Industries and Associations (EFPIA). Within PROMPT (PROMs suitable for assessing changes in acute postsurgical pain), one initiative (, work package 2 of IMI-PainCare) seeks to improve postoperative pain by effective perioperative pain management in terms of developing a COS of patient-reported outcome measures assessing efficacy and effectiveness in any clinical and observational studies as well as in clinical practice. Due to the assumable fact, that outcome domains and measurement instruments might differ dependently to specific treatment effects of certain surgical procedures (see for example www.postoppain.org, 30th March 2020), four surgical procedures are addressed: sternotomy (St), breast cancer surgery (BS), total knee arthroplasty (TKA), and surgery for endometriosis (EM). Those surgeries were chosen for (1) the differences in type and extent of tissue injury (soft tissue, bone/joint/ visceral), (2) the differences in patient populations (young versus old; cancer versus non-cancer patients, preoperative pain or none), (3) the frequency of practice and the concomitant moderate to severe postoperative pain, thus being relevant both for new treatment approaches and for many patients and health care practitioners worldwide. These procedures represent a broad spectrum of requirements of perioperative pain management, with the option to address the question of developing either separate COS for perioperative pain management after each surgical procedure or an overarching COS comprising all.

## Methods

### General considerations

**Fig. 1:**
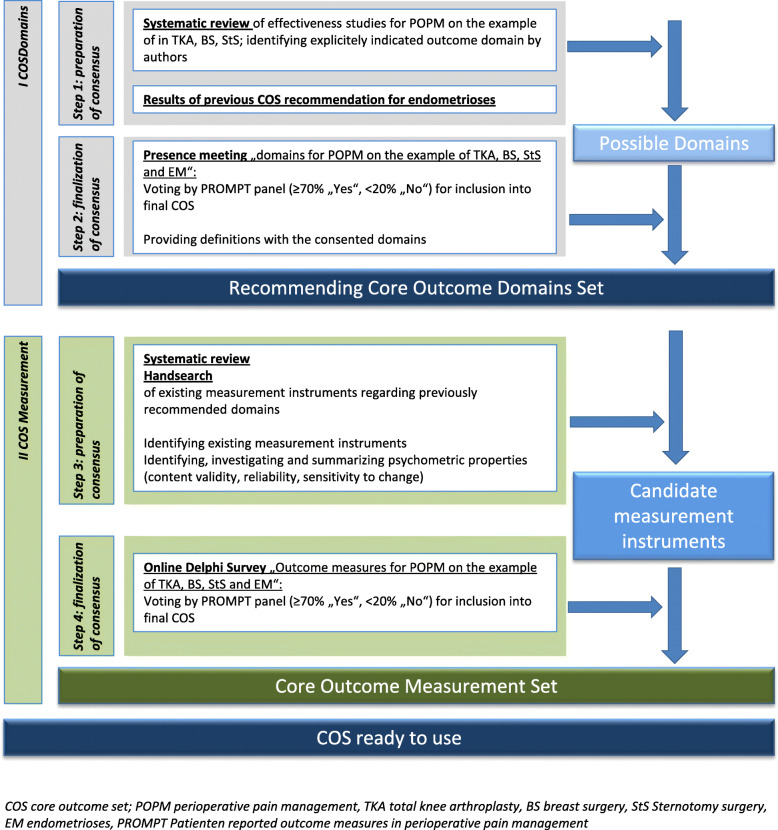
Schedule and Steps of the IMI PROMPT Consensus on a Core Outcome PROM Set for surgery after TKA, BS, ST and EM

Rationale and design for the consensus process within the PROMPT project are guided by the COMET recommendations ([12], see Fig. 1) referring to postoperative pain in adult patients undergoing breast surgery, sternotomy, total knee arthroplasty, and surgery for endometriosis and receiving perioperative pain management investigated in any clinical and observational trial as well as in clinical practice (scope), consisting of two parts—one for identifying core outcome domains and one for the corresponding core outcome measurement instruments. Both arms start with systematic literature research (SLR) and lead into different forms of consensus processes. Reporting of the processes will be basing on COS Star guidelines [35] (Fig. 1).

Ensuring comprehensiveness of a future COS in specific health conditions, theoretical frameworks should guide decision making about the systematization and the importance of core outcome domains ([12] Fig. 1, I COS Domains, steps 1 and 2). The framework recommended by OMERACT is matching purpose and need of PROMPT, providing a frame for medically oriented clinical trials and will be applied in order to arrange and structure relevant outcome domains to relevant core areas during the consensus meeting [36].

The focus on therapy aims as a prerequisite for deriving relevant outcome domains shall facilitate discussion and enable participants to be focused (Fig. 1, I COS Domains, step 2). Therapy aims of perioperative management after surgery (including BS, TKA, ST, and EM) contain restoration of impaired function (for example physical and/or psychological), the reduction of suffering due to postoperative pain considering a patient-centered approach, and the risks and benefits associated with the intervention within the first weeks after surgery (lay definition by the authors). According to the definition, an outcome domain (as a further specification of an aspect of health) and outcome (as any identified result in a domain arising from exposure to a causal factor or health intervention, modified from [36]) correspond closely to therapy intention or aim. Only aspects of health, changed by a specific intervention, seem to reasonably be considered as outcomes, because this change is intended and supposed to be investigated by comparative research. The close relationship between therapy aims and outcome domains will be consistently guided throughout the consensus process on outcome domains. An overview of the planned process for identifying COS of domains and measurement instruments for perioperative pain management after surgery in clinical trials and clinical practice is presented in Fig. 1.

For estimating and evaluating psychometric properties for future measurement instruments (Fig. 1, II COS Measurement, steps 3 and 4), standards have been established by the COSMIN working group. They advise to systematically search for evidence of psychometric properties of certain measurement instruments, defining clear criteria for good quality of study conduction *and* quality of scales. Psychometric properties of highest importance are validity (especially content validity as the prerequisite for further evaluation of other issues of scale quality), reliability, and sensitivity to change [37]. The COSMIN checklist also provides guidance on how to evaluate validity, reliability, and sensitivity to change in measurement instruments [38, 39].

### Participants of the IMI PROMPT consensus panel

For perioperative pain management, a considerable variety of stakeholders can be assumed, especially regarding the latest acceleration in treatment development and multi-professionality in this field. Ten different stakeholders have found to be relevant by the steering committee (see Table 1).

**Table 1:** PROMPT consensus panel

Stakeholder groups	Nominated by
Anesthesiologists	ESA ESRA
Pain specialists	IMI Group EFIC
IMI Group	IMI Group
Surgeons	EFIC IMI Group
Psychologists	EFIC IMI Group
Physiotherapists	EFIC IMI Group
HTA/PRO experts and regulatory experts	IMI Group
Pain nurses	EFIC IMI Group
Patient representatives	EFIC IMI Group
IMI-EFPIA	IMI Group

*ESA* European Society of Anaesthesiologists, *EFIC* European Pain Federation, *ESRA* European Society of Regional Anesthesia and Pain Therapy, *IMI Group*IMI-PainCare Consortium

Eligible for the IMI PROMPT consensus panel are individuals experienced in perioperative pain management (clinicians, researchers) after breast surgery, thoracotomy/sternotomy, total knee arthroplasty, and endometrioses or having experienced such procedure or other painful surgeries themselves (patient representatives). For endometriosis, inclusion was extended to individuals experienced in unspecified treatment of endometriosis. Further, pharma representatives, representatives of health technology assessment agencies, and regulators (experienced in drug development) have been identified as important stakeholders in the field of perioperative pain management. Since PROMPT is embedded into the large EU-funded project (please compare www.IMI-paincare.eu, 30th March 2020) and other processes will be basing on PROMPT future results, the IMI PROMPT consensus panel was extended by corresponding working group members of the IMI-PainCare project (comprising functional pain biomarkers (BioPain) and Translational Research in Pelvic Pain (TriPP)), announced by the IMI-PainCare leaders. In preparation of the process, international scientific organizations associated with pain research and management (EFIC) or anesthesia and postoperative pain management (ESA) have been approached and were invited to nominate at least 4 relevant experts from their field of interest. In Table 1, stakeholder groups are provided along with addressed scientific and patient self-help organizations nominating representatives for their participation as well.

Group size considerations referred to the consensus meeting because of financial, timely, and administrative resources on one side and requirements for equal distribution of stakeholder representatives on the other. During the consensus, four separate breakout groups have been planned (TKA, BS, St, and EM) with at least one stakeholder representative from each group (*n* = 10). It was therefore intended that each stakeholder group should consists of 4 representatives, in sum a number of *n* = 40 was expected to participate.

The same panel will be invited to the online Delphi survey on measurement instruments (Fig. 1, steps 2 and 4).

### Information sources

According to the COMET data source (http://www.comet-initiative.org/, 30th March 2020) and to the best of our knowledge, no COS initiative is engaged in perioperative pain management in general or for the chosen procedures total knee arthroplasty, breast surgery, and sternotomy so far.

Endometriosis is being worked on by several initiatives with different scopes, either published as report or study protocol [31, 33, 34, 40] or not published yet but provided to authors of this manuscript [personal communication with Katy Vincent, Email during June 2018]. Considering this work is required, thus, following COMET recommendation of careful consideration of previous work in the field of interest [12], it was therefore necessary to break down this consensus process into two different arms, at least referring to COS domains.

#### Initial list of outcome domains (Fig. 1, step 1)

An initial list of outcome domains for effectiveness assessment of perioperative pain management after TKA, BS, and St will be gathered via separate SLRs, all registered at PROSPERO database [CRD42018093838; CRD42018095142; CRD42018095137], where comprehensive details can be found. The searches will be conducted in Embase, MEDLINE, and CENTRAL (without timely or quantity restriction of publication) until 2018, searching for all forms of clinical prospective observational and randomized controlled trials regarding effectiveness of perioperative pain management after TKA, BS, and St. No quality assessment (e.g., by GRADE) is intended since the sole frequency of outcomes or outcome domains will be of interest. Screening of title/abstract and full text will be performed by two independent reviewers. Extraction will contain, besides study characteristics, types and frequencies of the applied outcomes or outcome domains forming a descriptive synthesis.

For endometriosis, previous results [31–34, 40] of initiatives specifically dedicated to study and improve treatment of endometriosis will be provided to the IMI PROMPT consensus panel after deciding about main aims of perioperative pain management in patients with endometriosis. This will be used in order to subsequently decide whether these recommended domains serve the purpose of capturing effectiveness in perioperative pain management in endometriosis or if modifications are needed. Key issues of discussion will be documented and reported in the future meeting report.

#### Initial list of measurement instruments (Fig. 1, step 3)

Based on the a priori defined and recommended outcome domains for perioperative pain management after surgery (BS, TKA, St, and EM), relevant measurement instruments will be identified via systematic review in two steps following the COSMIN guidance. The first step will comprise a scoping review and hand search for measurement instruments mapping outcome domains and definitions. Search will be performed in Embase, MEDLINE, and CENTRAL, in case of psychological domains additionally in PsychINFO and PsychArticle and, if needed, in common data bases for PROMs. Two independent reviewers will screen the results for title and abstract and for full text. Extraction will contain, besides study characteristics, definition of construct, description of the developmental process (in terms of identifying patient-reported outcome measures), scale construction, description of scale, and preliminary results in case of first validation. Primary search terms will refer to inauguration articles (articles describing the development of a scale or measurement instrument) and the specific domain (including synonyms or related terms).

COSMIN and COMET suggest searching also in their database of SLRs regarding measurement instruments. In case of old or low-quality SLRs, they advise to conduct an update or to perform a new SLR; otherwise, it is considered sufficient to rely on existing results.

Measurement instruments corresponding to IMI PROMPT outcome domain definitions, developed as patient-reported outcome and with a similar target population (postoperative, acute pain; similar characteristics as observed in TKA, BS, St, and EM) will be chosen for further investigation of psychometric properties regarding the COSMIN quality criteria for creating a list of potential PROMs.

COSMIN search strings will be applied [41] for subsequent systematic literature reviews in the abovementioned data sources, concerning at least one of the psychometric properties regarding content validity, construct validity, reliability, and sensitivity to change. For both validities, studies will be included when presenting results for construct and/or content validity. For reliability and sensitivity to change, studies will be included when providing information to the a priori defined target population (adult patients undergoing surgery for BS, TKA, St, and EM). Quality approval includes quality of study conduction and reporting and, in a second step, quality of the scale, performed by COSMIN checklist [38, 39, 42, 43]. All information for each scale will be finally summarized in a table providing the definition of the construct, the identified results regarding validity (content and construct), reliability, and sensitivity to change, including quality ratings of study quality and quality of the psychometric property separately. The final tables will then be provided to the IMI PROMPT consensus panel during the online Delphi survey for further decision about relevant measurement instruments.

### Consensus process

#### Outcome domains- consensus meeting

The consensus process (a 1-day face to face consensus meeting) will be facilitated by two members of the steering committee (EPZ, UK). A structured schedule (provided as a handout), consisting of plenary discussions, breakout groups (stakeholder groups, groups working on the specific health conditions) shall support consistent and transparent discussion and approaching a stable consensus.

Enhancing respectful discussion, the IMI PROMPT consensus panel will be advised to discuss from the perspective of their stakeholder group, not as unique person, wherefore a basic understanding of representative of a stakeholder group will be formed by a specific, introductory part. Brainstorming and aligning on therapy aims and finally choosing relevant corresponding outcome domains for perioperative pain management will be performed within breakout groups referring to each surgery separately. All results of the breakout groups will be discussed subsequently after completing each step (aligning on therapy aims, aligning on corresponding outcome domains) and overarching results, comprising perioperative pain management in all surgery groups in general, are appreciated. Members of the steering committee (HL, WM, PZ, and CW) will facilitate the breakout groups. The variety of steps and groups enables equal chances to contribute for each participant.

The first section of the meeting aims for the aligning on relevant and most critical therapy aims of perioperative pain management. Starting with a brainstorming on relevant therapy aims for each surgery (via world café, 4 rounds), all participants will be invited to leave as much information to the breakout groups as they feel is relevant. A prioritization within the breakout groups on most critical therapy aims will complete this section. The breakout groups prioritize therapy aims according to the OMERACT 2.0 filter ([36], four core areas: death, life impact, resource use/economical impact, pathophysiological manifestations, adverse events) and also according to their relevance on a 1–9 scale (Likert scale, 1–3 not important, 4–7 important but not critical, 7–9 critical). Presenting the breakout group results to the plenary group will invite comprehensive feedback of other participants. The IMI PROMPT consensus panel will also be encouraged to structure therapy aims either into general therapy aims of perioperative pain management or into more specific therapy aims regarding surgery in the four regions.

During a subsequent section, outcome domains from systematic literature review (initial list of outcomes) will be matched to the a priori aligned most critical therapy aims (rated as critical (7–9)), added by official definitions of those outcome domains if available. Regarding alignment on relevant and important therapy aims, existing and via SLR-identified outcome domains can be included, excluded, or merged into each other, always ensuring transparent documentation of the process. Reasons for excluding, merging, or otherwise amending outcome domains will be reported in the future meeting report. The group also will have the opportunity to decide either to choose generic (for perioperative pain management in general) or specific (for perioperative pain management after the specific surgery) outcome domains. In case of competing outcome domains within one therapy aim, the panel will approve the outcome domain matching most and drop the less important outcome domain regarding the therapy aim.

It will be suggested to recommend at least one domain for each core area; otherwise, it will be explicitly explained why a core area has not been considered for COS [12].

In a final plenary section (complete IMI PROMPT consensus panel), all breakout groups present and discuss their results. When discussion is completed, the voting will be performed for all outcome domains ranging from 7 to 9 as most critical, starting with generic suggestions. Four outcome domains are expected. The outcome domain with the highest rating will be preferred in case of competing outcome domains.

Patient representatives are able to veto in case of complete disagreement with a single outcome domain. Patient representatives need to be unanimously against a decision of other stakeholder groups to set a veto. If another stakeholder group consistently disagrees with a panel decision, it is able to advice the panel to discuss the issue again, but the panel needs to approve the necessity of that action. In case of disapproval to discuss an outcome domain again, further discussion will be dismissed and the group will move forward to the next outcome domain.

#### Outcome measurement instruments-Delphi online exercise

Consensus on measurement instruments shall be achieved via a final online Delphi survey (Surveymonkey), planned as a 3-step online survey. Information resulting from a second set of systematic literature reviews, now on psychometric properties of corresponding measurement instruments (PROMs) and search for construct definition of the relevant measurement instruments will be provided to the IMI PROMPT consensus panel (see “Participants of the IMI PROMPT consensus panel”), alongside with quality grading by COSMIN checklist for both study and scale quality. Each member of the IMI PROMPT consensus panel participants will be invited to comment and to preliminarily vote for each presented instrument on 1–9 scales as described above, advised to focus on highest available quality. Summarizing results from this first round regarding all presented measurement instruments, added by summarized feedback by panel participants, the preliminary vote of the complete IMI PROMPT consensus panel and of the individuals will be presented during a second round, also inviting comments and feedback to each instrument. The final vote in the third round will only include those measurement instruments which have been rated to be sufficient for inclusion (rating of 7–9 on the 1–9 scale) into future COS by at least 50% of participants in at least 2/3 of stakeholder groups.

### Scoring of outcome domains and measurement instruments

Considering the quality of reporting outcome domains in intervention and effectiveness studies in terms of perioperative pain management after BS, TKA, St, and EM, only few studies have reported outcome domains explicitly and clearly defined. During the consensus meeting, outcome domains will be classified into 1–3 not important, 4–6 important but not critical, 7–9 critical, as recommended by COMET [12]. Inclusion of outcome domains requires an outcome domain to be rated as at least 7 on the 1–9 rating scale.

The same scoring will be applied for measurement instruments during an online Delphi survey. In case of competing measurement instruments for a single outcome domain, the measurement instrument with the highest rating will be included into future COS.

### Consensus definition

Consensus on outcome domains (consensus face to face meeting) and measurement instruments (online Delphi exercise) will be defined as at least ≥70% voting for 7–9 and ≤ 20% voting for 1–3 rating (means that most of the panel feels that the specific outcome domain is important to include) by the IMI PROMPT consensus panel in order to include an outcome domain and a measurement instrument into future COS (see Table 2), adapted by COMET recommendation of 70% vs 15% [12] for reasons of feasibility. Exclusion is defined as 70% of voting for 1–3 and 20% voting for 7–9 for a certain outcome domain or measurement instrument. All other cases will be considered no consensus, which means that there is uncertainty about the importance of the specific outcome domain or measurement instrument to be included into future COS.

**Table 2:** Definition of consensus for PROMPT consensus on outcome domains and measurement instruments regarding perioperative pain management in patients after surgery (BS, TKA, St, and EM) [REF HARMAN 2013]

Consensus classification	Description	Definition
Consensus “in”	Consensus that either outcome domain or measurement instrument should be included into COS for perioperative pain management after surgery (BS, TKA, St, and EM)	70% or more participants scoring 7–9 AND 20% or less participants scoring 1–3
Consensus “out”	Consensus that either outcome domain or measurement instrument should NOT be included into COS for perioperative pain management after surgery (BS, TKA, St, and EM)	70% or more participants scoring 1–3 AND 20% or less participants scoring 7–9
No consensus	Uncertain about importance of outcome	Anything else

### Project schedule

- Systematic reviews on outcome domains 11/2017–06/2018 (completed, publication of results is in preparation or submission, DATE OF SUBMISSION)
- Consensus meeting on outcome domains 06/2018 (completed, publication of results in in preparation)
- Systematic reviews and research between 08/2018 and 08/2020 (SLRs are submitted to PROSPERO, search strategy is completed, screening of title/abstract and full text is completed for scoping review on corresponding measurement instruments, SLR for psychometric properties is in preparation).
- Preparation of results of systematic reviews for psychometric properties of corresponding measurement instruments and preparation of the online Delphi exercise between 09/2020 and 09/2021
- Final consensus on outcome measurement instruments between 09/2021 and 03/2022

## Discussion

As summarized in previous publications [44], there are many ways for establishing a COS on domains so far. Yet, the Delphi method is one of the most accepted. This method is characterized by the opportunity to avoid biases in response, decision, or opinion building, easily restricting dominant stakeholder representatives [45] and supporting to include individuals worldwide [12]. Consensus methods have been used as well, but there is no clear evidence of superiority of specific methods [12]. Both ways are recommended, and sometimes they are applied together in mixed method approaches.

Major limitations of both approaches as forms of qualitative processes comprise the affection of the final results by types of stakeholders involved, the existing knowledge of the participants [46], the questions asked, the provision of information (or false information [46]), and the manner of interaction [45]. A careful consideration of the planned rationale is therefore necessary, balancing field of future COS, financial and timely resources, the amount of stakeholder groups necessary, and the complexity of the scope, aim, and domains to be considered of the future COS [12]. Since a standardization of methods cannot be recommended to date for COS development, detailed and precise reporting of conduction is demanded [12, 35].

For Delphi methods, additionally attrition during the iterative process is highly relevant, challenging process completion and interpretation of results, especially when missing specific stakeholder groups [46]. The complexity of the questions addressed might be strained by the format of repeating exposure to similar looking questionnaires and produces effects of tiredness and motivation loss [46]. All those aspects endanger reliability and representativity of Delphi processes, even though when conducted in large samples internationally. As discussed by other authors [47], psychometric soundness of consensus processes (e.g., Delphi) containing estimates for reliability, validity, and generalizability might not serve properly. They suggest rather estimating quality of such processes, considering the qualitative field of research, by other criteria such as transferability, credibility, applicability, or confirmability of results. They explicitly state that such process do not aim for finding the right answer but support orientation and create impulses in research and health care provision.

Based on these experiences and the situation of heterogeneity in designing COS developmental studies, the steering committee of IMI PROMPT decided to apply a mixed method approach for establishing the COS for perioperative pain management in four surgeries for clinical trials. One main consideration started with the observation that bringing together different stakeholders with different backgrounds in an online survey does not support understanding and acceptance since backgrounds and opinions cannot be exchanged easily. There is a considerable risk that results of such consensus repeat what has been there already, not assuming or critically reflecting on still missing aspects. Solely relying on outcome domains already published also creates biases, based on individual routines or preferences. Besides the limits of face to face meetings in timely and financial resources, it indeed provides all participants with much more information by discussion and exchange. The application of a facilitating schedule (guided discussion, breakout groups, plenary sessions) was intended to reduce the danger of potential biases due to opinion leading by single individuals or strong stakeholder groups enhancing acceptance, transparency, and presence of multiple perspectives. Since developing COS domains refers much to personal backgrounds, needs, and preferences, a face to face meeting was considered most appropriate and, at the end, time saving. For defining COS on measurement instruments, performing an online based Delphi survey will suffice, since there are several distinct criteria (quality of study conduction, quality of scale-related psychometric properties), facilitating preparation of online Delphi survey and decision by the panel.

There are possible limitations to our schedule and design. One refers to the representativeness of the panel. The announcement by organizations might help to reduce the selection bias induced by possible preferences of the steering committee, but which representatives will be chosen depends much on the organization. The steering committee has no control of real expertise, motivation, and background of the participants. Patients will be announced also by an international patient advocacy organization in order to send patients experienced in consensus processes and sufficiently speaking English. Since international networking of patient advocacy organizations is still a work in progress, selection bias for patients cannot be ruled out, such as coming from countries with a high amount of well-educated inhabitants, culturally open and experienced in scientific discussions.

Complexity of consensus processes and the preparation of such time- and resource-consuming multifaceted approaches for developing COS might be a discouragement. Yet, there is no alternative referring to the major aim of finding best ways for improved care for our patients.

## Data Availability

Data will be available on request of authors.
